# An Oil-Bath-Based 293 K to 473 K Blackbody Source

**DOI:** 10.6028/jres.101.062

**Published:** 1996

**Authors:** Joel B. Fowler

**Affiliations:** National Institute of Standards and Technology, Gaithersburg, MD 20899-0001

**Keywords:** aperture, blackbody, cavity, conical, emissivity, radiation, radiometry, reflectance, source, temperature, thermometer, oil bath

## Abstract

A high temperature oil-bath-based-black-body source has been designed and constructed in the Radiometric Physics Division at the National Institute of Standards and Technology, Gaithersburg, MD. The goal of this work was to design a large aperture blackbody source with highly uniform radiance across the aperture, good temporal stability, and good reproducibility. This blackbody source operates in the 293 K to 473 K range with blackbody temperature combined standard uncertainties of 7.2 mK to 30.9 mK. The calculated emissivity of this source is 0.9997 with a standard uncertainty of 0.0003. With a 50 mm limiting aperture at the cavity entrance, the emissivity increases to 0.99996.

## 1. Introduction

The oil bath based blackbody source described in this paper was developed as a large area source of highly uniform and stable thermal radiation for the calibration of infrared detectors, radiation thermometers and thermal imaging systems with relatively large fields of view. The cavity has a wide (10.8 cm) diameter aperture and an extended conical cavity section similar to the earlier generations of water-bath-based designs described in Refs. [1] and [6] which operate in the 293 K to 353 K source temperature range. This new oil-bath-based blackbody source extends this temperature range to approximately 473 K with excellent source temperature uniformity and stability. The oil temperature stability of this source is ±2 mK or less over many days; the temperature uniformity of the oil volume is ±2 mK at the lowest temperature in its operating range and ±8 mK at the high end of its operating range of 293 K to 473 K, as measured using the resistance thermometry detailed later in this paper.

## 2. Design

This blackbody source incorporates a Hart Scientific Model 6024^1^ temperature-controlled bath with GPIB control capability (specially modified by Hart Scientific to accept the cavity) to heat the oil in which the cavity is immersed and a Hart Scientific electronic thermometer Model 1575 used in conjunction with a Hart Scientific platinum resistance thermometer model 5682 to measure accurately the temperature of the oil in the bath. Hereafter, the model 6024 bath will be referred to as the bath, the model 1575 electronic thermometer as the electronic thermometer, and the model 5682 SPRT as the PRT probe unless otherwise specified.

The heating source is laminated into a single plate covering the bottom of the bath well along with an external cooling loop. The oil in the bath well is agitated by a low speed double stirrer. The cavity is mounted in the side of the bath. Dow Corning 200.20 silicone oil is used as the bath medium.

### 2.1 Cavity Design and Construction

The cavity was constructed using oxygen-free copper. The conical portion was machined from a solid round bar and the cylindrical portion was machined from a section of tubing to 0.005 cm wall thickness. These two parts, along with a 6.35 mm thick mounting ring, were then brazed together in a vacuum oven using a high- copper-content-alloy brazing material. The oxygen-free copper has a very high thermal conductivity of 3.88 W/(cm·K) [2], which improves the thermal uniformity of the cavity and decreases the thermal resistance of the cavity wall. The outer surface of the cavity was plated with a thin layer of gold over nickel to retard oxidation of the copper surface.

**Fig. 1:**
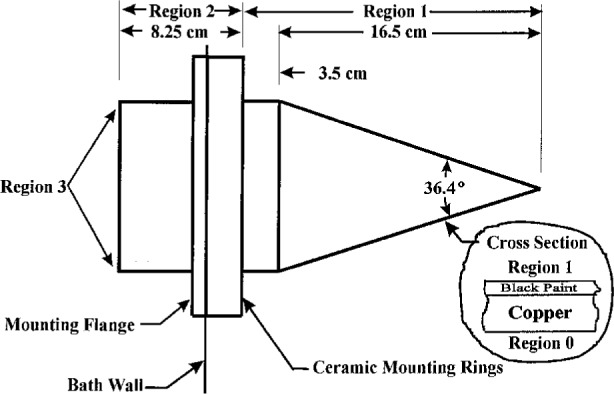
Schematic layout of the cavity details.

High temperature paint was applied to the interior surface of the cavity wall. The cavity was first lightly sanded inside with 600 grit wet-dry sandpaper lubricated with ethyl alcohol and cleaned first with acetone and finally with Dampney thinner. The cavity was then set in a ring stand with the tip of the cavity down and approximately 10 mL of Dampney Thurmalox 2102–30 high gloss heat resistant paint was introduced into the cavity. The cavity was then rotated axially while being slowly tipped from vertical to horizontal, thereby coating the entire interior surface with the paint. Once coated, the cavity was placed on a sheet of paper with the tip pointing up, thereby permitting the excess paint to drain out of the cavity while at the same time keeping the paint fluid by trapping the vapors from the paint inside the cavity. Once the excess paint had drained out of the cavity, in about 2 h, the cavity was supported approximately 12 mm above the paper and the paint permitted to harden. Support in this manner reduces the chance of distortion of the paint due to changes in the force acting on the paint due to gravity and keeps dust away from the inside cavity surface while the paint is hardening. The paint was tack free and firm after 2 days. The cavity was then baked at 150 °C for 8 h. Useful dimensions and other properties of the cavity section are shown in Fig. 1.

The cavity was sealed into the front vertical wall of the bath using two ceramic rings and high temperature silicone adhesive.

## 3. Control and Measurement

The bath electronics control the heating cycle through the use of a proportional-integral-derivative (PID) control loop. The heating control circuitry allows achievement of bulk oil temperature stability of better than ±2 mK with the cavity installed. The temperature of the oil in the bath is measured with the electronic thermometer in conjunction with the PRT probe. The electronic thermometer contributes a temperature measurement uncertainty^2^ of 1 mK and the PRT probe contributes an additional temperature measurement uncertainty of less than 1.0 mK. The temperature set-point of the bath is controlled by, and the temperature data from the electronic thermometer is read by, a digital computer utilizing a GPIB interface and software developed at NIST.

The bath may be controlled by either control panel settings or the GPIB interface. The method of setting the temperature is similar either way. When under program control, the desired temperature is input to the program. The bath is then commanded by the program to go to the desired temperature setpoint in the coarse setting mode which has a 1 K setting accuracy. The bath temperature is repeatedly read using the electronic thermometer. When the temperature has stabilized close to the set-point, with an instability of ±10 mK or less, the bath is commanded in the high resolution setting mode (which is capable of setting the bath temperature relative to the measured bath temperature to better than ±1.0 mK) to change the temperature by the difference between the desired temperature and the actual measured temperature. After this final temperature setpoint adjustment, the bath will attain a temperature setpoint well within the requirements of this application. The computer program monitors and logs the temperature of the bath continuously while at this setpoint.

An external chiller is required to supply the internal cooling coil in the bath with 278 K water at setpoints below 353 K in order to maintain a sufficient cooling load to the bath to maintain the required stability. Unlike the past water-bath-based sources [6], the PID (proportional, integral, derivative) proportionality band and heater level must be also be changed for optimum control of the bath temperature. The optimum PID settings are listed in Table 1 for 10 setpoint temperatures.

## 4. Temperature Measurement

### 4.1 Measurement of the Oil-Volume Thermal Uniformity Around the Cavity

The temperature uniformity of the oil volume surrounding the cavity wall was measured with the electronic thermometer, using two PRT probes, one on each of the two input channels of the instrument. One PRT probe was positioned at a fixed reference point near the cavity tip and the other PRT probe was movable around the perimeter of the cavity. The movable PRT probe was positioned at ten locations, at three different levels around the outline of the cavity using a special ceramic fixture. The three levels correspond to the centerline of the cavity, bottom edge of the cavity, and top edge of the cavity. Immersion effects were minimized by the design of the fixture which enclosed the upper portion of the movable PRT probe with hot air or oil vapor; thereby reducing the immersion loss errors to a low level.

These measurements indicate that the maximum temperature deviation at each of the three levels around the periphery of the cavity varies from 1 mK near the cavity tip to 3 mK near the cavity wall at 293 K, varies from 2 mK near the cavity tip to 7 mK near the cavity wall at 373 K, varies from 2 mK near the cavity tip to 13 mK near the cavity wall at 433 K, and varies from 2 mK near the cavity tip to 14 mK near the cavity wall at 473 K referenced to the fixed probe. The highest values of the deviations of the oil temperature at each of the above four temperatures were chosen as the conservative values of the oil temperature non-uniformity at those temperatures. The worst case temperature non-uniformity values chosen to be used in the calculations are given in Table 2.

### 4.2 Cavity Lip Temperature

A differential thermocouple thermometer was used to measure the temperature drop between the immersed cavity components and the outside cavity lip. One thermocouple junction was imbedded in white heat sink compound and pressed against the outer lip of the cavity with a 20 cm length of its cable wrapped tightly around the outer cavity lip. The junction of the other thermocouple was pressed against the wetted portion of the conical section of the cavity with a mechanical clamp while immersed in the bath oil with approximately a 20 cm length of its cable immersed in the oil near the probe. Each channel of the differential thermometer was calibrated using a bath whose temperature was measured and set with the Hart thermometer to near 393.00 K. Differences ranging from 0.2 K at the 293 K setpoint to 32.6 K at the 473 K setpoint were measured between the cavity lip and the immersed tip of the cavity. Both channels were then rechecked at the conclusion of the measurement. The standard uncertainty of the differential thermometer was 50 mK, as both indicated in the manufacturer’s data sheet and verified by the electronic thermometer.

### 4.3 Oil Temperature Uncertainties

The oil temperature uncertainties can be divided into two categories. The first is the combined contribution from the external temperature measurement system consisting of the electronic thermometer and the PRT probe, along with the possible temperature control errors due to the bath temperature control characteristics. The second consists of the uncertainties due to the thermal properties of the cavity, the effects of the cavity interior coating, and environmental effects due to such things as convection, stray air currents, etc.

The uncertainties associated with the control and external measurement of the temperature of the oil in the bath are straightforward in that only the contributions from the oil bath temperature control and the electronic thermometer-PRT probe combination need to be considered. The bath control circuitry contributes an absolute temperature setting uncertainty of 1 K without external temperature measurement and less than 1.0 mK with external temperature measurement when using the high-resolution-setting mode as stated by the manufacturer. The external temperature measurement uncertainty is due to the PRT probe and the electronic thermometer. The PRT probe contributes 1.0 mK uncertainty as calibrated at NIST. The Hart electronic thermometer contributes an uncertainty of 1 mK or less as calibrated by the factory and traceable to a NIST standard, resulting in a combined standard uncertainty of 1.4 mK for the combination of the electronic thermometer and the PRT probe. The uncertainty in the bath temperature stability is due to the bath control loop which has an instability of less than ±2 mK and has no contribution from the external thermometry for a given setpoint temperature within the operating range of the instrument. Table 3 shows the uncertainties associated with the temperature of the oil in the bath.

The combined standard uncertainty of the bath oil temperature may be calculated by adding in quadrature the standard uncertainties associated with the oil temperature non-uniformity, bath temperature setting error, possible PRT probe immersion error, temperature measurement error due to the PRT probe, and temperature measurement error due to the electronic thermometer, yielding a standard uncertainty of 7.2 mK at 293 K, a standard uncertainty of 9.6 mK at 353 K, a standard uncertainty of 18.2 mK at 433 K, and a standard uncertainty of 18.2 mK at 473 K using values shown in Tables 2 and 3. These are worst case standard uncertainties of the oil temperature at any point in the bath surrounding the cavity, but do not take into account the interface between the oil and the cavity, and the thermal characteristics of the cavity. These last two factors are taken into account in the calculations described later in this paper.

These blackbody sources will generally be used with a lower cost Hart Scientific Model 1506 electronic thermometer with a temperature measurement standard uncertainty of 6 mK, as stated by the manufacturer and referenced to a NIST standard when used in combination with the PRT probe (same type used with the model 1575 thermometer). The substitution of the model 1506 thermometer will increase the combined standard uncertainty of the oil temperature measurement to 9 mK. The more expensive model 1575 thermometer was used in these tests only to determine how stable and accurate this blackbody source could be with high accuracy components.

## 5. Cavity Emissivity

Ideally, when the walls of the cavity are in local thermodynamic equilibrium, the emissivity of the cavity *ϵ* is 1 minus the cavity reflectance. Using this fact, we may calculate an approximation for the emissivity *ϵ* based on the assumption that the reflectance of the interior wall of the cavity is the sum of a perfectly specular component *ρ*_s_ and a perfectly diffuse component *ρ*_d_. The specular reflectance is divided into two components: one to account for the specular reflectance at normal incidence and one to account for the specular reflectance at lower angles of incidence. This is necessary, as the angle of incidence of each reflection after entering the cavity varies. For radiation entering the cavity at near normal incidence, the emissivity *ε* is expressed by [3]

$$\varepsilon =1-(\rho_{\text{ss}}^{2}\rho_{\text{sh}}^{2})-dF_{13}\rho_{d},$$ (1)

where d*F*_13_ is the differential configuration factor which describes the fraction of the radiation emitted from a differential area d*A* on the cavity wall which exits through the opening of the cavity; *ρ*_sh_ is the specular reflectance of the cavity surface at high angles of incidence for the first two reflections; *ρ*_ss_ is the specular reflectance of the cavity surface at smaller angles of incidence for the remaining two reflections; and *ρ*_d_ is the diffuse reflectance of the paint. We have ignored the small variation of *ε* with wavelength *λ*. Four reflections inside the cavity were chosen due to physical limitations of the bath and the manufacture of the cavity and diminishing returns from additional reflections. These four reflections produce an absorption of 99.999 % of the radiation entering the cavity. Equation (1), though not a worst case approximation, may be used for a worst case analysis by choosing conservative estimates for *ρ*_d_, *ρ*_sh_ and *ρ*_ss_.

The measured reflectance of a “witness” sample of the black gloss coating used inside the cavity and applied to the same copper material from which the cavity was machined is 4 % total reflectance up to 10 µm, 8 % up to 20 µm, and rises rapidly past 20 µm at near normal incidence. The specular reflectance value is increased from 4 % to 8 % up to 10 µm and 8 % to 16 % at 20 µm for the larger angles of incidence. Though no data were available for the diffuse reflectance beyond 2.5 µm, the diffuse reflectance is known to be < 0.2 % between 800 nm and 2.5 µm and becomes less diffuse and more specular with increasing wavelength. The reflectance for this cavity was chosen to be a conservative value of *ρ*_sh_ = 16 % specular for the larger angles of incidence, *ρ*_ss_ = 8 % specular for the smaller angles of incidence, and *ρ*_d_ = 0.2 % for the diffuse reflectance. The value of the differential configuration factor d*F*_13_ was calculated for nominal cavity dimensions and varies from the tip of the conical section of the cavity to the cylindrical-conical intersection. A typical value of d*F*_13_ = 0.03 was chosen.

Utilizing the above values, Eq. (1) yields 0.9997 for a lower bound for the emissivity *ε* with 0.9997 ± 0.0003 for a conservative estimate for the spectral range of 1 µm to 30 µm at a blackbody temperature of 473 K. If a 50 mm diameter aperture with high infrared reflectance on the side facing the cavity were added to the front of the cylindrical portion of the cavity and the calculation repeated, the emissivity increases to near 0.99996 at normal incidence to the cavity. As a check of the above calculations, the emissivity was recalculated utilizing the EE31 computer program written by Prokhorov and Sapritsky [3] for the calculation of black-body emissivity. For the same parameters as used in the above calculations, this computer program yields an effective emissivity of 0.9998 at normal incidence. Recalculating with the addition of a 50 mm aperture in front of the cylindrical portion of the cavity, the emissivity normal to the cavity increased to 0.99990. These values represent the worst case situation of a temperature setpoint of 473 K. This program also can account for nonuniform temperature distributions over the cross sectional and longitudinal dimensions of the cavity. Randomly varying the temperature uniformity input to the computer program by as much as ±100 mK, a much worse case than our maximum measured nonuniformity, the normal emissivity calculated was never less than 0.9991 without the 50 mm aperture or less than 0.99991 with the 50 mm aperture. This confirms our assumption that the nonuniformity of the surface temperature of the cavity as measured in this instrument is not significant.

Effects such as air currents and the consequences of off-axis viewing have been ignored and will be addressed in a report on the radiometric testing of the blackbody currently being performed at NIST.

## 6. Temperature Drop Across the Cavity Wall and Paint

### 6.1 Temperature Distribution Over the Interior Cavity Surface

The worst case approximations used to estimate the temperature drops in regions 0 and 1, as shown in Fig. 1, for this the cavity are as follows:

1. Region 3 is at a uniform temperature near ambient throughout (*T*_3_) assuming negligible conduction along the cavity wall.
2. Region 2 is at a uniform temperature throughout (*T*_2_).
3. The worst case value for the temperature in region 2 is the temperature at the very edge of the cavity lip.
4. Region 0, the surface in contact with the bath oil, is at a uniform temperature *T*_0_ which is the same temperature as the bath oil.

For high accuracy measurements, the temperature of the bath oil must be very stable and accurately measured. The oil in this bath was accurately measured for stability and absolute temperature as outlined in Sec. 4.1 and meets this requirement in excess of the extent necessary to achieve the desired quality of the source. The term quality will be described later.

### 6.2 Temperature Drop Across the Cavity Wall and the Black Paint

The differential heat conduction across the cavity wall in region 1 and radiating out of the cavity is given by

$$dP=(T_{0}-T_{1})/(d_{\text{cu}}/k_{\text{cu}}+d_{\text{bp}}/K_{\text{bp}}),$$ (2)

where *d*_cu_ is the cavity wall thickness, *K*_cu_ is the thermal conductivity for the copper wall of the cavity, *d*_bp_ is the thickness of the paint, and *K*_bp_ is the thermal conductivity of the paint. For the assumed thermal equilibrium, the above quantity must balance the net differential radiant power leaving the surface of the paint on the inside of the cavity wall at any point in region 1 as shown in Fig. 1. This quantity d*P* is given by

$$dP=dF_{13}\;\sigma (T_{1}^{4}-T_{3}^{4})+(dF_{12}-dF_{13})\sigma (T_{1}^{4}-T_{2}^{4})$$ (3)

where *σ* is the Stefan-Bolzmann constant, and d*F_ij_* is the differential configuration factor from the point of interest in region *i* to all of region *j*. Because the temperature difference between *T*_0_ and *T*_1_ is small, the error introduced by approximating $T_{1}^{4}$ by

$$T_{1}^{4}=T_{0}^{4}+4T_{0}^{3}\;\Delta T$$ (4)

is negligible, where ∆*T* = *T*_1_ – *T*_0_ is the temperature drop across the cavity wall and paint. Equations (3) and (4) may be solved simultaneously for *T* in closed form:

$$\Delta T=\frac{-\beta T_{0}(dF_{13}[1-{\left(T_{3}/T_{0}\right)}^{4}]+[dF_{12}dF_{13}][1-{\left(T_{2}/T_{0}\right)}^{4}]}{1+4dF_{12}\beta }$$ (5)

where

$$\beta =\sigma T_{0}^{3}(d_{\text{cu}}/K_{\text{cu}}+d_{\text{bp}}/K_{\text{bp}}).$$ (6)

Table 4 enumerates the nominal values used in the evaluation of ∆*T* in Region 1. The temperature of region 3 was chosen to be nominally 295 K. The above analysis is similar to the analysis presented in NBS Technical Note 1228 [1] and has been modified to reflect the changes in the design of this blackbody.

The paint thickness was measured by taking the difference between the thickness of the coated metal blank used for the “witness” sample in the measurement of the reflectance of the black paint before and after coating. Although the method of coating the sample was performed to approximate closely the inside of the cavity, the estimate of 0.005 cm may be incorrect by up to 50 % therefore *d*_bp_ = (0.005 ± 0.0025) cm has been chosen as a conservative estimate.

Values for the temperature drop ∆*T* across the cavity wall at the intersection of the conical and cylindrical portions of the cavity that are totally immersed in the temperature controlled oil were calculated and are shown in Table 5 for several oil temperatures, along with the associated uncertainties using the worst case parameters for each value. Table 6 shows the resulting combined standard uncertainty for the temperature of the interior of the cavity.

## 7. Blackbody Quality

The blackbody quality accounts for the effects of temperature gradients between the oil in the bath and the cavity surface, and the cavity wall reflectance in a single quantity [4, 5]. Quality is defined here in terms of a reference temperature, which is conveniently the temperature which is actually being measured during the operation of the blackbody, the oil temperature in this case. It is the ratio of two radiances that are important: the actual cavity radiance, and the ideal Planck-law radiance at this reference temperature.

A simple expression for the quality of a blackbody of this type can therefore be expressed by [1]

$$Q=\varepsilon [\exp(c_{2}/\lambda T_{0})-1]/[\exp(c_{2}/\lambda T)-1]$$ (7)

where *ε* is the emissivity (again ignoring the small variations of *ε* with *λ*), *λ* is the wavelength of interest, *T*_0_ is the ideal (reference) temperature, *T* is the actual (effective) cavity temperature and *c*_2_ is the second radiation constant. *Q* is simply the calculated emissivity modified by the ratio of the ideal and actual Planck law radiances.

Taking the first two terms of a Taylor series expansion of the right hand side of Eq. (7) and substituting ∆*T* = *T* – *T*_0_ when the second term is small compared to unity, Eq. (7) may be approximated [1] by

$$Q\approx \varepsilon [1+(\Delta T/T_{0})(c_{2}/\lambda T_{0})/(1-\exp(-c_{2}/\lambda T_{0}))].$$ (8)

### 7.1 Overall Blackbody Quality

We can use Eq. (8) to calculate the quality of the blackbody at any wavelength and to calculate the relative combined standard uncertainty of the quality using the values calculated for ∆*T* and *ε*.

An equation for the relative combined standard uncertainty in the blackbody quality is [1]

$$u_{c,r}(Q)=\sqrt{Q[{\left(u(\varepsilon )/\varepsilon \right)}^{2}+{\left(F(c_{2}/\lambda T_{0})u(T)/T_{0}\right)}^{2}]},$$ (9)

where

**Fig. 2:**
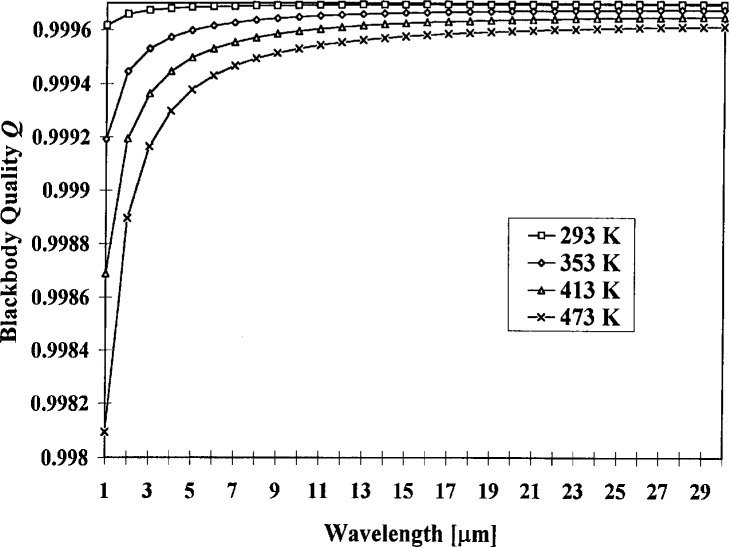
Blackbody quality as a function of wavelength for four different temperatures *T*.

**Fig. 3:**
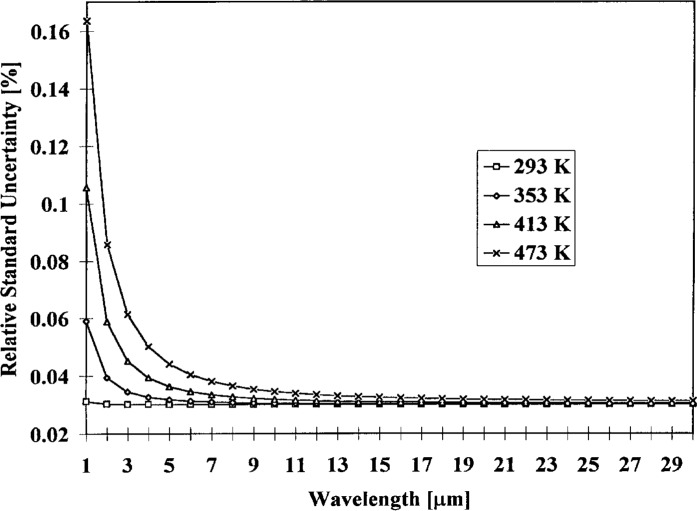
Blackbody quality relative standard uncertainty as a function of wavelength.

F(x)⋅x/(1−exp(−x)). (10)

and may be derived from Eq. (7). Table 7 shows the relevant values used in the calculation of the quality and the uncertainty of the quality, and Figs. 2 and 3 graph the quality and it’s uncertainty versus wavelength. (Quality is a method for comparing blackbody designs.)

## 8. Conclusion

A high quality thermometer was used in the evaluation and in the operation of the oil bath during testing. In the normal use of this instrument, a thermometer only slightly better than the expected performance need be used. The Hart Scientific Model 1506 electronic thermometer with a PRT probe suits this need very nicely. Substitution of the Model 1506 only degrades the performance by the increased uncertainty of the thermometer. The temperature measurement combined standard uncertainty of the Model 1506 electronic thermometer when used in conjunction with the PRT probe is 6.1 mK compared to 1.4 mK for the model 1575 electronic thermometer used with the same PRT probe. The blackbody quality *Q* would only decrease by 0.1 % of *Q* at long wavelengths and 0.01 % of *Q* at the shorter wavelengths if used with the lower accuracy thermometer.

The design meets our expectations whether used with the Hart Super Thermometer or the Model 1506 Metrology thermometer. The calculated emissivity is very high and we expect excellent radiometric characteristics. Radiometric measurements are currently being conducted at NIST and will be the subject of a future paper.

## Figures and Tables

**Table 1:** Proportionality bandwidth settings, heater settings, and chiller requirements for 10 temperature setpoints

Temperature setpoint(K)	Proportionality bandwidth	Heater low/high	Chiller in/out
293	0.101	low	in
313	0.101	low	in
333	0.101	low	in
353	0.101	low	out
373	0.151	low	out
393	0.199	low	out
413	0.199	low	out
433	0.407	high	out
453	0.407	high	out
473	0.407	high	out

**Table 2:** Oil temperature non-uniformity and standard uncertainty values used in the uncertainty analysis at ten set-point temperatures

Oil temperature (K)	Nonuniformity (mK)	Standard uncertainty (mK)
293	−1 to +2	3
313	−1 to +3	4
333	0 to −7	7
353	0 to −7	7
373	0 to −18	18
393	0 to −19	19
413	0 to −19	19
433	0 to −17	17
453	0 to −17	17
473	0 to −17	17

**Table 3:** Oil temperature measurement and control errors and standard uncertainties used in uncertainty analysis

Source of uncertainty	Range of possible temperature error values (mK)	Standard uncertainty (mK)
Electronic thermometer	±1.0	1.0
PRT probe	±1.0	1.0
Stability of bath	±2.0	2.0
Bath setting	±1.0	1.0
Estimated insertion loss	±6.0	6.0

**Table 4:** Values of parameters used to calculate ∆*T* from Eq. (5)

Parameter	Value
Cavity internal diameter	10.7 cm
Length of cylindrical cavity section	10.9 cm
Full angle of cavity	38 °
Thickness of cavity wall	0.4 cm
Thickness of black paint	0.005 cm
Thermal conductivity of cavity wall	3.8 W/(cm·K)
Thermal conductivity of black paint	0.0018 W/(cm·K)
d*F*_12_	.052
d*F*_13_	.03

**Table 5:** Calculated maximum temperature drop across the cavity wall from the bath oil to the painted surface inside the cavity denoted above as region 1 (nominal ambient temperature 295 K)

Oil temperature *T*_0_ (K)	Temperature drop ∆*T* (mK)	Standard uncertainty (mK)
293	−0.2	0.2
313	−1.0	1.0
333	−2.6	2.6
353	4.4	4.4
373	6.6	6.6
393	9.2	9.2
413	12.0	12.0
433	15.8	15.8
453	20.0	20.0
473	25.0	25.0

**Table 6:** Total combined standard uncertainty of the temperature of the interior of the cavity

Oil temperature (K)	Uncertainty due to oil temperature nonuniformit (mK)	Uncertainty due to oil temperature measurement and control (mK)	Uncertainty due to to temperature drop across cavity wall (mK)	Total combined standard uncertainty of the temperature of the cavity interior *u*_c_(*T*) (mK)
293	3	6.5	0.2	7.2
313	4	6.5	1.0	7.7
333	7	6.5	2.6	9.9
353	7	6.5	4.4	10.6
373	18	6.5	6.6	20.3
393	19	6.5	9.2	22.1
413	19	6.5	12.0	23.4
433	17	6.5	15.8	24.1
453	17	6.5	20.0	27.0
473	17	6.5	25.0	30.9

**Table 7:** Parameters used in the calculation of the blackbody quality and uncertainty using Eq. (1) and Eqs. (8) and (9)

∆*T* (mK)	Standard uncertainty *u*(∆*T*)^a^ (mK)	*T*_0_ (K)
−0.2	7.2	293
−1.0	7.7	313
−2.6	9.9	333
−4.4	10.6	353
−6.6	20.3	373
−9.2	22.1	393
−12.0	23.4	413
−15.8	24.1	433
−20.0	27.0	453
−25.0	30.9	473

*ε*	Standard uncertainty *u*(*ε*)	Restrictions

0.9997	0.0003	No aperture
0.99996	0.00004	50 mm aperture

a These values are only valid when viewing the conical section of the cavity.

## Footnotes

1 Certain commercial equipment, instruments, or materials are identified in this paper to foster understanding. Such identification does not imply recommendation or endorsement by the National Institute of Standards and Technology, nor does it imply that the materials or equipment identified are necessarily the best available for the purpose.

2 All uncertainties given in this paper are standard uncertainties (i.e., 1 standard deviation estimates) unless otherwise specified.
